# Supplementary material for: Mesozooplankton grazing minimally impacts phytoplankton abundance during spring in the western North Atlantic
Source: PeerJ. 2020 Jul 17;8:e9430. doi: 10.7717/peerj.9430 (PMC7370934; doi:10.7717/peerj.9430)
Supplement: Supplemental Information 1 — Abundance is given as number of individuals per m3. [file peerj-08-9430-s001.docx]

| **Abundance (indiv. m^-3^)** | **S1** | **S2** | **S4** |
| --- | --- | --- | --- |
| *Calanus finmarchicus* | 6.6 | 2535.2 | 828.5 |
| *Calanus hyperboreus* | 3.4 | 0.0 | 0.0 |
| *Metridia lucens* | 0.3 | 60.4 | 27.2 |
| *Pseudocalanus* spp. | 0.0 | 0.0 | 115.4 |
| *Paracalanus* spp. | 0.0 | 0.0 | 556.8 |
| *Pleuromamma* | 0.0 | 0.0 | 88.3 |
| Other | 0.0 | 150.9 | 400.6 |
