# Supplementary material for: Mesozooplankton grazing minimally impacts phytoplankton abundance during spring in the western North Atlantic
Source: PeerJ. 2020 Jul 17;8:e9430. doi: 10.7717/peerj.9430 (PMC7370934; doi:10.7717/peerj.9430)
Supplement: Supplemental Information 2 — Light had a significant effect on net phytoplankton growth rates, accounting for 75.76 % of the total variance, whereas the effect of grazer type was not significant (7.36% of total variance). The interaction between the two factors was not significant (see text for detail). [file peerj-08-9430-s002.docx]

| **Source of variation** | **SS (Type III)** | **DF** | | **MS** | **F (DFn, DFd)** | **P value** |
| --- | --- | --- | --- | --- | --- | --- |
| Predator type | 0.0200 | 1 | 0.0200 | | F (1, 6) = 3.63 | P= 0.10 |
| Light | 0.2060 | 1 | 0.2060 | | F (1, 6) = 37.39 | P< 0.01 |
| Interaction | 0.0004 | 1 | 0.0004 | | F (1, 6) = 0.08 | P= 0.79 |
| Residual | 0.0331 | 6 | 0.0055 | |  |  |
