# Supplementary material for: Mesozooplankton grazing minimally impacts phytoplankton abundance during spring in the western North Atlantic
Source: PeerJ. 2020 Jul 17;8:e9430. doi: 10.7717/peerj.9430 (PMC7370934; doi:10.7717/peerj.9430)
Supplement: Supplemental Information 3 — Apparent (net) phytoplankton growth rates (k, d −1) in the nutrient-amended (+N) 20% and 100% ¡200 μ m seawater dilutions used to quantify microzooplankton grazing rates.Rates shown are based on 24 h changes in chlorophyll a and are given per day, ± one standard deviation of the mean of triplicate measurements. [file peerj-08-9430-s003.docx]

| **Station** | **Replicate** | **20% + N** | **100% + N** |
| --- | --- | --- | --- |
| 1 | A | 0.38 (0.10) | 0.02 (0.04) |
| 1 | B | 0.36 (0.05) | 0.16 (0.05) |
| 2 | A | 0.84 (0.06) | 0.70 (0.26) |
| 2 | B | 0.79 (0.07) | 0.63 (0.28) |
| 4 | A | 0.40 (0.16) | 0.58 (0.11) |
| 4 | B | 0.43 (0.11) | 0.63 (0.11) |
