# Supplementary figures and images for: Mesozooplankton grazing minimally impacts phytoplankton abundance during spring in the western North Atlantic
Source: PeerJ. 2020 Jul 17;8:e9430. doi: 10.7717/peerj.9430 (PMC7370934; doi:10.7717/peerj.9430)

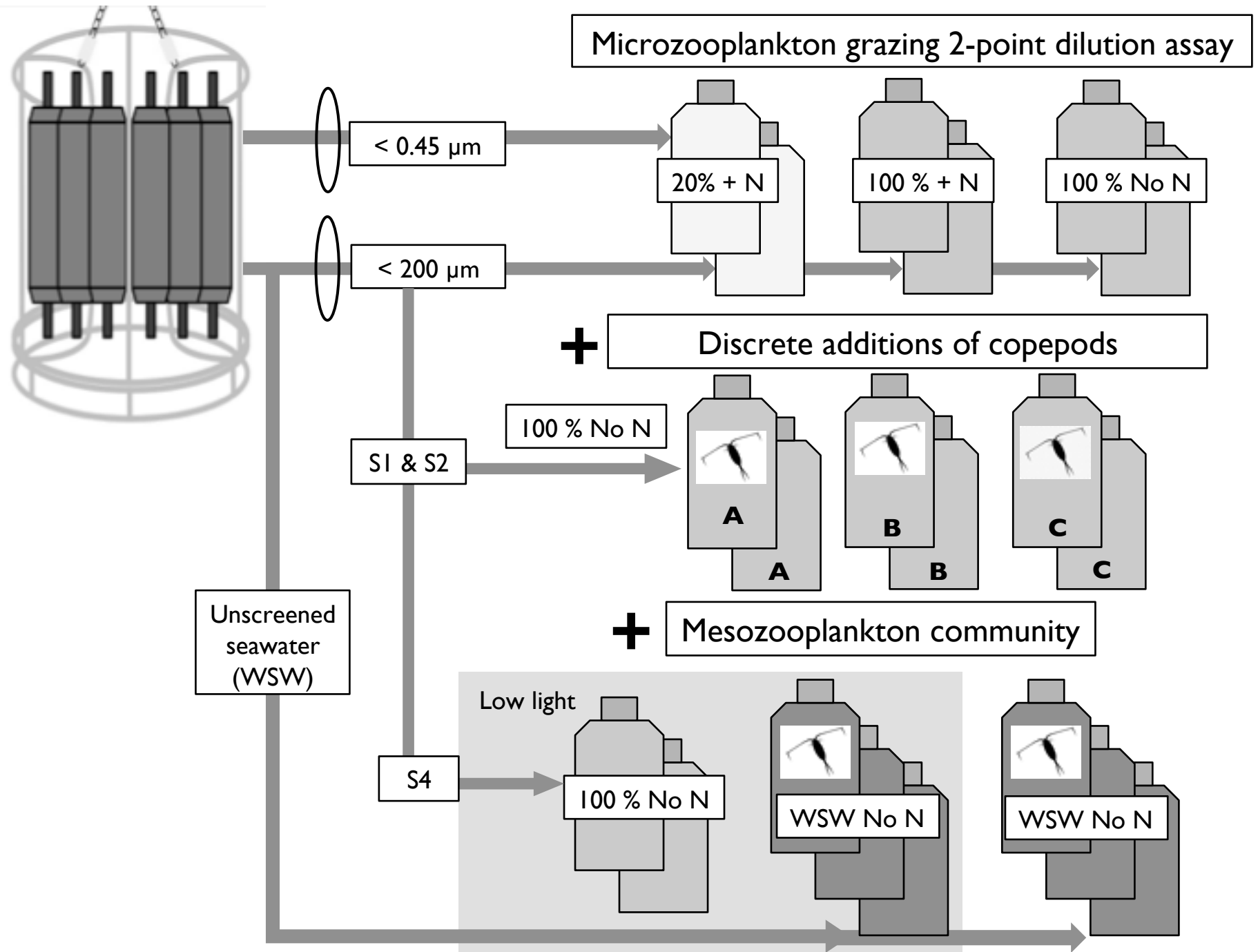

Supplement: Supplemental Information 4 — Multiple treatments with discrete additions of copepods were conducted at two stations, with A, B, C corresponding respectively to the addition of 1, 3, and 5 copepods at S1 and 10, 20, and 40 copepods at S2. Unscreened seawater was used at S4 to represent the mesozooplankton treatment. At S4, the mesozooplankton treatment and control were incubated both at in situ and reduced light intensity. [file peerj-08-9430-s004.pdf]
